# Community socioeconomic disadvantage drives type of 30-day medical-surgical revisits among patients with serious mental illness

**DOI:** 10.1186/s12913-021-06605-y

**Published:** 2021-07-05

**Authors:** Hayley D. Germack, Khadejah Mahmoud, Mandy Cooper, Heather Vincent, Krista Koller, Grant R. Martsolf

**Affiliations:** 1 University of Pittsburgh School of Nursing, 3500 Victoria Street 336 Victoria Building, 15261 Pittsburgh, PA USA; 2 University of Pittsburgh Graduate School of Public Health, 130 De Soto Street, 15261 Pittsburgh, PA USA; 3 Community College of Allegheny County, 710 Duncan Avenue, 15237 Pittsburgh, PA USA; 4 University of Pittsburgh College of General Studies, 1400 Wesley W. Posvar Hall 230 S. Bouquet St, 15260 PA Pittsburgh, USA; 5 RAND Corporation, 4570 Fifth Ave #600, 15213 Pittsburgh, PA USA

**Keywords:** Revisits, Readmissions, Observation stays, Socioeconomic disadvantage, Mental illness

## Abstract

**Background:**

Patients with serious mental illness (SMI) are vulnerable to medical-surgical readmissions and emergency department visits.

**Methods:**

We studied 1,914,619 patients with SMI discharged after medical-surgical admissions in Florida and New York between 2012 and 2015 and their revisits to the hospital within 30 days of discharge.

**Results:**

Patients with SMI from the most disadvantaged communities had greater adjusted 30-day revisit rates than patients from less disadvantaged communities. Among those that experienced a revisit, patients from the most disadvantaged communities had 7.3 % greater 30-day observation stay revisits.

**Conclusions:**

These results suggest that additional investments are needed to ensure that patients with SMI from the most disadvantaged communities are receiving appropriate post-discharge care.

**Supplementary Information:**

The online version contains supplementary material available at 10.1186/s12913-021-06605-y.

## Background

People with serious mental illness (SMI; i.e., schizophrenia, major depressive disorder, and bipolar disorder) are at increased risk of morbidity and mortality compared to the general population [1, 2]. In fact, SMI is associated with nearly 8 years of reduced life expectancy [2], attributable to the high prevalence of diabetes, cardiovascular disease and hypertension in this population [3, 4]. While researchers often focus on psychiatric hospitalizations for people with SMI, it is critical to attend to their higher than average medical hospitalizations. People with SMI have higher rates of inpatient hospitalization [5, 6] and emergency department (ED) visits [7]—contributing to their four times greater healthcare expenditures [8]. They are particularly vulnerable to revisits (i.e. unplanned returns to the hospital within 30 days of discharge including ED visits and readmissions [unplanned admissions to medical-surgical units]) after medical-surgical hospitalizations given pronounced barriers in engaging in positive health behaviors [9, 10].

The empirical literature has documented associations between readmissions among adults with SMI and the communities that they are discharged to. One study identified a higher likelihood of 1-year psychiatric readmission among Hispanic patients, patients with at least one prior hospitalization, patients discharged to a location near a Narcotics Anonymous meeting place, and residing in an area with low educational attainment [11]. Another study found that community residential mobility (i.e. a combination of the percentage of residents living in a different house in the past year and the percentage of non-owner occupied housing) was significantly associated with fewer mental health visits even after controlling for other community-and individual-level factors among people with SMI and comorbid medical illness [12]. Many studies have established that people who live in socioeconomically disadvantaged communities (i.e. communities challenged by low income, limited education, and substandard living conditions) [13] have worse mental and physical health outcomes than people living in more socioeconomically advantaged communities [14, 15]. People with SMI often live in disadvantaged communities [16]. However, previous research has not extensively explored the community context which this population returns to after discharge from medical-surgical hospitalizations [17, 18]. Community-level factors can influence health outcomes independently of commonly measured individual-level factors, through pathways such as exposure to chronic stress, lack of access to proper follow up care (e.g. no local primary care availability), and lack of healthy food availability (e.g. no stores that offer health food in the communities), each of which may exacerbate existing conditions and increase the likelihood of readmission [19, 20].

Research investigating the association between community socioeconomic disadvantage and readmissions has increased in recent years, but these studies largely ignore the other types of acute, unscheduled post-discharge revisits [13, 21]. Patients can also return to the emergency department (ED) or they may be admitted on observation units, without requiring a formal readmission (i.e. an inpatient stay) [22]. Observation stays are replacing some readmissions, making it important to capture observation stays as a type of revisit [23]. There are significant gaps in our understanding of how community socioeconomic disadvantage impacts revisits to the emergency department and observation stays, particularly among people with SMI, who are largely impacted by the community in which they live. Understanding the relationship between community socioeconomic disadvantage and different types of medical-surgical revisits (ED visits, observation stays, and readmissions) in this population is necessary as researchers pay greater attention to the relationship between social determinants of health and health outcomes including unscheduled post-discharge care.

Given what is known about the complex mechanisms that drive revisits, examining the association between community socioeconomic disadvantage and medical-surgical revisits among patients with comorbid SMI is important. Understanding the community socioeconomic context which people return to is critical because post-discharge follow-up occurs within a geographic area (e.g. county, community) and community factors summarized by community socioeconomic disadvantage are associated with greater risk of 30-day readmission in the general population [21]. This study used four years (2012–2015) of discharge data from two large states (Florida and New York) to examine whether community socioeconomic disadvantage was associated with 30-day medical-surgical revisits in patients with SMI after adjusting for patient and hospital-level characteristics.

## Methods

### Database and Study Population

We examined acute care utilization for adults (age ≥ 18 years) using Healthcare Cost and Utilization Project (HCUP) 2012–2015 discharge data from Florida and New York from the State Inpatient Database (SID), State Emergency Department Database (SEDD), and State Ambulatory Surgery and Services Database (SASD) from the Agency for Healthcare Research and Quality (AHRQ). The SID, SEDD, and SASD are all-payer databases, containing discharges from nonfederal, non-psychiatric community hospitals and emergency departments. They contain more than 100 clinical and nonclinical variables including principal and secondary diagnoses and procedures, admission and discharge status, patient demographics, and length of stay (LOS) [24, 25]. These de-identified data are available to researchers for a nominal fee through the AHRQ website. Florida and New York comprise 12 % of the US population and their discharge data had encrypted patient identification numbers that permitted linkage across facilities and hospitals. The years 2012–2015 were selected because they occurred after the Hospital Readmission Reduction Program took effect and before International Classification of Diseases-10 (ICD-10) changes in coding were implemented.

We combined patient-level data from the 2012–2015 SID, SEDD, and SASD with hospital-level data from the 2015 American Hospital Association (AHA) Annual Survey, community-level data from the 2015 Area Health Resources Files (AHRF) and with ZIP-level data for 2015 from the University of Wisconsin Neighborhood Atlas® Area Deprivation Index (ADI) [26]. The 2015 AHA Annual Survey provided data on hospital structural and organizational characteristics. The AHA data are collected annually from over 6,400 U.S. hospitals and are available for purchase from the AHA [27]. The 2015 AHRF data are available for download from the Health Resources and Services Administration website [28]. The 2015 ADI data are available for download from the ADI website [29].

We included all non-psychiatric medical-surgical admissions from HCUP. Index admissions for patients aged 18 years and older were considered if they occurred at nonfederal general medical-surgical hospitals. Using AHRQ Clinical Classification Software (CCS) codes, all discharge records for patients with comorbid diagnoses of SMI were identified (Supplementary File 1). Patients were excluded from the cohort if they: (1) did not survive to discharge; (2) had an admission with a primary diagnosis of SMI; (3) had an index length of stay of less than or equal to one day; (4) discharged against medical advice; and (5) were admitted for a diagnosis category or a procedure considered planned (i.e. primary diagnosis of cancer or procedures including obstetrical delivery, transplant surgery, maintenance chemotherapy, rehabilitation; ICD9 and AHRQ CCS categories used to identify planned admissions in the Supplementary File 1) [30].

### Measures

#### Community Socioeconomic Disadvantage

2015 data from the University of Wisconsin Neighborhood Atlas® ADI [26] were used to characterize patients’ community socioeconomic disadvantage. The ADI allows for rankings of neighborhoods by socioeconomic status disadvantage in a region of interest (e.g. at the state or national level). It uses American Community Survey (ACS) Five Year Estimates in its construction. The 2015 ADI used the ACS data for 2015, which is a 5-year average of ACS data obtained from 2011 to 2015. It includes factors from the theoretical domains of income, education, employment, and housing quality. In the present study, ADIs were aggregated to the ZIP-level for communities in Florida and New York. Communities were compared independently for Florida and New York—the 50 % least disadvantaged communities were grouped together; the 45 % middle disadvantaged communities were grouped together; and the 5 % most disadvantaged communities were grouped together.

#### Revisit Types

The revisit was defined as the first revisit for a physical health condition within 30 days of discharge. For the purpose of this study, revisit types include ED visits, observational stays and readmissions. The primary outcomes of interest were three types of medical-surgical revisit: (1) having an ED visit within 30-days of discharge; (2) having an observation stay within 30 days of discharge; and (3) having a readmission within 30 days of discharge. An additional outcome included having any type of revisit within 30 days of discharge (i.e. ED visit or observation stay or readmission). ED visits, observation stays, and readmissions were identified using the HCUP supplemental variables for revisit analysis, which provide a unique visit link to allow for each patient to be tracked at subsequent inpatient visits across time and institutions [31].

### Statistical Analysis

Patient, hospital, and community characteristics related to readmissions were included in the analyses. Individual patient-level demographic characteristics related to readmissions and included in our analyses were age, sex, primary payer (Medicare, Medicaid, private, self-pay, no charge, or other [32–35]. Patient clinical characteristics related to readmission included length of stay of the index admission, admission type (emergency, urgent, elective, or trauma center), an indicator for if they had a surgical procedure, Elixhauser comorbidity readmission risk score, and DRG of the index admission [32–35]. Hospital-level characteristics related to readmissions included teaching status of the hospital (member of Council of Accredited Teaching Hospitals), total number of hospital beds, technology status of the hospital (i.e. capable of performing heart transplant or adult interventional cardiac catheterization), the hospital’s nurse-to-bed ratio, and the ownership status of the hospital (i.e. non-federal government, private for profit, or private not-for-profit) [36–44]. Community characteristics related to readmissions included rurality, health care provider supply (ratio of primary care physicians to county population and ratio of nurse practitioners to county population) [45–47]. Patient, hospital, and community characteristics were compared by community socioeconomic disadvantage category (least 50 % disadvantaged; middle 45 % disadvantaged; and 5 % most disadvantaged) using chi-squared tests for categorical variables and analysis of variance for continuous variables.

Multivariate logistic regression models with state and year fixed effects were used to examine the relationship between a patient’s community socioeconomic disadvantage category and revisits (any revisit and by revisit type) after adjusting for patient, hospital, and community-level factors. Among patients who experienced a revisit, a second set of logistic regression models were used to examine the relationship between community socioeconomic disadvantage and revisit type after adjusting for patient, hospital, and community-level factors. Post-estimation commands were used to estimate the adjusted revisit rates of each type of revisit by community socioeconomic disadvantage. Statistical analyses were performed using STATA statistical software, version 16.1 (StataCorp, College Station, TX). For all analyses, *p* values of < 0.05 were considered statistically significant.

## Results

As presented in Table 1, patients’ demographic and clinical characteristics differed significantly between patients living in communities characterized by different levels of socioeconomic disadvantage. Patients whose primary residence was in the most disadvantaged communities were on average, nearly 5 years younger than those from the most advantaged communities (53.7 years vs. 58.5 years, *p* < 0.001). A greater proportion of patients from the most disadvantaged communities were female (52.4 % vs. 50.3 %: *p* < 0.001). There was also a significant difference in the distribution of patients based on primary payer status—among patients from the most disadvantaged communities, Medicaid paid 33.9 % of admissions compared to 16.4 % from the least disadvantaged communities (*p* < 0.001).

**Table 1:** Patient Demographic and Clinical Characteristics, by Community Socioeconomic Disadvantage; Florida and New York, 2012-2015

	All patients	Least Disadvantaged	Middle 45% Disadvantaged	Top 5% Disadvantaged	*p*-value
Demographics	*N*= 1,915,039	*n*=958,196	*n*=865,655	*n*=91,188	
Age (mean, SD)	57.7, 17.1	58.5, 17.5	57.2, 16.8	53.7, 16.1	<0.001
Age (N, %)					<0.001
18-44	421,194, 22.0%	203,676, 21.3%	192,651, 22.3%	24,867, 27.3%	
45-64	802,649, 41.9%	385,388, 40.2%	373,659, 43.1%	43,602, 47.8%	
65+	691,196, 36.1%	369,132, 38.5%	299,345, 34.6%	22,719, 24.9%	
Female (N, %)	987,148, 51.6%	481,688, 50.3%	457,694, 52.9%	47,766, 52.4%	<0.001
Primary Payer (N, %)					<0.001
Medicare	867,429, 45.3%	430,697, 45.0%	401,793, 46.4%	34,939, 38.3%	
Medicaid	341,919, 17.9%	156,762, 16.4%	154,258, 17.8%	30,899, 33.9%	
Private	460,920, 24.1%	270,892, 28.3%	177,315, 20.5%	12,713, 13.9%	
Self-Pay	140,339, 7.3%	56,227, 5.9%	76,614, 8.9%	7,498, 8.2%	
No Charge	30,713, 1.6%	10,220, 1.1%	18,750, 2.2%	1,743, 1.9%	
Other	73,719, 3.9%	33,398, 3.5%	36,925, 4.3%	3,396, 3.7%	
**Clinical Characteristics**					
Length of Stay, days (mean, SD)	4.8, 5.1	4.8, 5.0	4.7, 5.0	5.2, 6.3	<0.001
Admission Type (N, %)					<0.001
Emergency	1,439,740, 75.2%	720,846, 75.3%	646,159, 74.7%	72,735, 79.8%	
Urgent	152,640, 8.0%	68,358, 7.1%	79,382, 9.2%	4,900, 5.4%	
Elective	311,958, 16.3%	164,786, 17.2%	134,218, 15.5%	12,954, 14.21	
Trauma Center	10,281, 0.5%	3,954, 0.4%	5,757, 0.7%	570, 0.6%	
Surgical admission (N, %)	958,196, 50%	274,237, 28.62%	233,221, 26.9%	21,163, 23.21%	<0.001
Elixhauser comorbidity readmission risk score (mean, SD)	12.5, 11.7	11.9, 11.5	13.1, 11.9	14.5, 12.5	<0.001

Note: *SD* Standard Deviation

There were also significant differences in patients’ clinical characteristics based on their community’s socioeconomic disadvantage. Compared to patients from the middle and least disadvantaged communities, patients from the most disadvantaged communities had longer lengths of stay (5.2 days vs. 4.7 and 5.8; *p* < 0.001), were more likely to be admitted for emergency reasons (as opposed to elective) (79.8 % vs. 74.7 %, and 75.3 %; *p* < 0.001), and had a higher Elixhauser comorbidity readmission risk score (14.5 vs. 13.1, and 11.9; *p* < 0.001). Supplementary File 2 displays that across all three categories of community disadvantage, patients had similar reasons for admission—the most common being cardiac (17.9 %), followed by nervous system (7.2 %), COPD/asthma (7.0 %), alcohol/drug (5.7 %), and renal failure (4.5 %).

As presented in Table 2, hospital characteristics differed significantly between patients living in communities characterized by different levels of socioeconomic disadvantage. Greater proportions of patients from the least and most disadvantaged communities received care in teaching hospitals (29.7 and 24.9 % vs. 17.5 %; *p* < 0.001). A greater proportion of patients from the most disadvantaged communities received care hospitals with a high technology status (78.4 % vs. 65.4 and 67.5 %, *p* < 0.001). Furthermore, a greater proportion of patients from the least and most disadvantaged communities received care in private, not-for profit hospitals (74.6 and 71.2 % vs. 59.2 %; *p* < 0.001).

**Table 2:** Hospital Characteristics, by Community Socioeconomic Disadvantage; Florida and New York, 2012-2015

Characteristic	All patients	Least Disadvantaged	Middle 45% Disadvantaged	Top 5% Disadvantaged	*p*-value
	*N*= 1,915,039	*n*=958,196	*n*=865,655	*n*=91,188	
Teaching Hospital (N, %)	458,359, 23.9%	284,508, 29.7%	151,149, 17.5%	22,702, 24.9%	<0.001
Bed Size (mean, SD)	551.1, 501.0	557.1, 484.3	529.0, 518.7	697.5, 475.4	<0.001
Bed Size, category (N, %					
6-24	2,322, 0.1%	569, 0.1%	1,694, 0.2%	59, 0.1%	<0.001
25-49	8,320, 0.4%	1,681, 0.2%	5,857, 0.7%	782, 0.9%	
50-99	79,460, 4.2%	36,083, 3.8%	40,373, 4.7%	3,004, 3.3%	
100-199	267,292, 14.0%	118,027, 12.3%	144,324, 16.7%	4,941, 5.4%	
200-299	346,423, 18.1%	159,656, 16.7%	177,074, 20.5%	9,693, 10.6%	
300-399	252,688, 13.2%	148,950, 15.5%	97,913, 11.3%	5,825, 6.4%	
400-499	219,120, 11.4%	90,412, 9.4%	114,630, 13.2%	14,078, 15.4%	
500+	739,414, 38.6%	402,818, 42.0%	283,790, 32.8%	52,806, 57.9%	
High technology status (N, %)	1,284,471, 67.1%	646,806, 67.5%	566,200, 65.4%	71,465, 78.4%	<0.001
Number of RNs per Bed (mean, SD)	1.5, 0.6	1.6, 0.6	1.4, 0.6	1.4, 0.6	<0.001
Ownership (N, %)					<0.001
Nonfederal, government run	228,417, 11.9%	118,441, 12.4%	96,935, 11.2%	13,041, 14.3%	
Private, not-for-profit	1,291,570, 67.4%	714,434, 74.6%	512,260, 59.2%	64,876, 71.2%	
Private, for-profit	395,052, 20.6%	125,321, 13.1%	256,460, 29.63%	13,271, 14.6%	

Note: *SD* Standard Deviation; *RN* Registered Nurse

As presented in Table 3, community characteristics differed substantially by community socioeconomic disadvantage. Compared to patients from the least disadvantaged communities, those from the most disadvantaged communities were more likely to be in rural counties (76 % vs. 74.6 %; *p* < 0.001). Additionally, they were from counties with fewer primary care physicians (251/10,000 county population vs. 299/10,000 county population; *p* < 0.001), and more nurse practitioners (69 per 10,000 county population vs. 56 per 10,000 county population; *p* < 0.001).

**Table 3:** Community Characteristics, by Community Socioeconomic Disadvantage; Florida and New York, 2012-2015

Characteristic	All patients	Least Disadvantaged	Middle 45% Disadvantaged	Top 5% Disadvantaged	*p*-value
	*N*= 1,915,039	*n*=958,196	*n*=865,655	*n*=91,188	<0.001
Rural County (N, %)	1,451,410 (75.8)	714,750 (74.6)	667,354 (77.1)	69,306 (76)	<0.001
Ratio of Primary Care Physicians per 10,000 Persons in County (mean, SD)	266 (178)	299 (181.1)	230 (171.1)	251 (108.6)	<0.001
Ratio of Nurse Practitioners per 10,000 Persons in County (mean, SD)	59.1 (32.3)	56.1 (31.9)	61.3 (31.9)	69.1 (37.7)	<0.001

Note: *SD* Standard Deviation; *RN* Registered Nurse

**Fig. 1:**
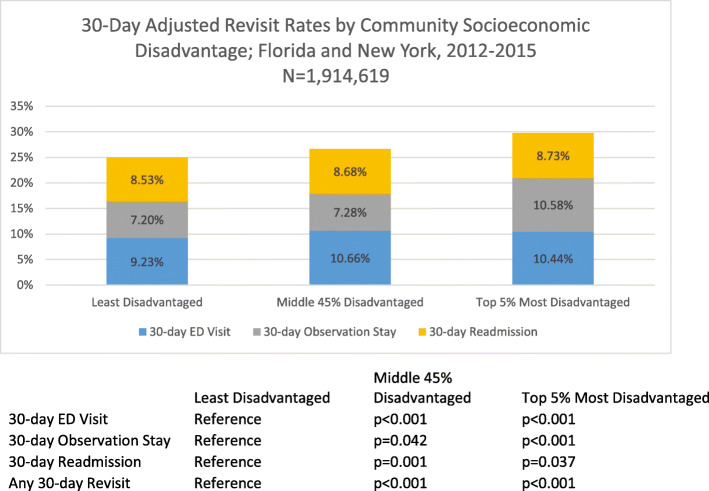
Note: Adjusted revisit rates based on logistic regressions models with least disadvantaged communities as the reference category after controlling for individual patient-level demographic (i.e. age, sex, primary payer [Medicare, Medicaid, private, self-pay, no charge, or other]) and clinical characteristics (length of stay of the index admission, admission type [emergency, urgent, elective, or trauma center] an indicator for if they had a surgical procedure, Elixhauser comorbidity readmission risk score, and DRG of the index admission) and hospital-level characteristics (teaching status of the hospital [member of Council of Accredited Teaching Hospitals], total number of hospital beds, technology status of the hospital [i.e. capable of performing heart transplant or adult interventional cardiac catheterization], the hospital’s nurse-to-bed ratio, and the ownership status of the hospital [i.e. non-federal government, private for profit, or private not-for-profit]); ED = Emergency Department

Figure 1 displays the post-estimation adjusted 30-day revisit rates by community socioeconomic disadvantage (full regression models with odds ratios shown in Supplementary File 3). The adjusted revisit rates were 30.33 % among patients in the most disadvantaged communities, 25.06 % in the middle 45 % of disadvantaged communities, and 25.94 % in the least disadvantaged communities. There were significant differences in the distribution of revisit type. The adjusted ED revisit rate was 10.44 % (*p* < 0.001) among patients in the most disadvantaged communities compared to 10.66 % (*p* < 0.001) in the middle disadvantaged communities, and 9.23 % in the least disadvantaged. The adjusted observation stay revisit rate was 10.58 % (*p* < 0.001) among patients from the most disadvantaged communities, 7.28 % (*p* = 0.042) in the middle disadvantaged communities and 10.58 % in the most disadvantaged communities. The adjusted readmission revisit rate was 8.73 % (*p* = 0.037) among patients in the most disadvantaged communities compared to 8.68 % (*p* < 0.001) in the middle 45 and 8.53 % in the least disadvantaged communities (*p* < 0.001).

**Fig. 2:**
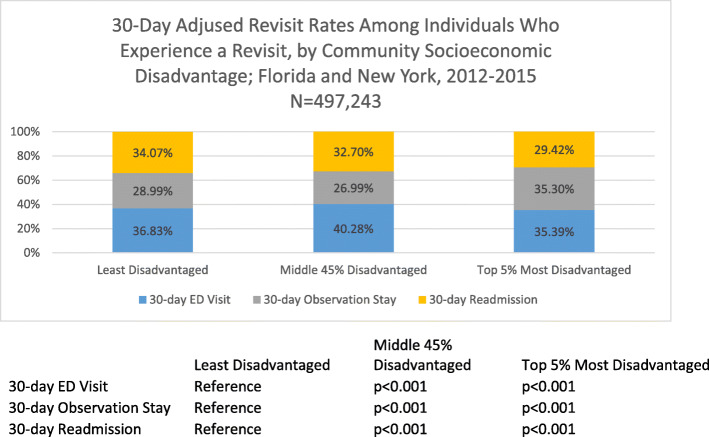
.

Figure 2 displays the post-estimation adjusted 30-day revisit rates among individuals who experience a revisit, by community socioeconomic disadvantage. The adjusted ED revisit rate was 35.39 % (*p* < 0.001) among the most disadvantaged, 40.28 % (*p* < 0.001) among the middle 45 %, and 36.83 % among the least disadvantaged. The adjusted observation stay revisit rate was 35.30 % (*p* < 0.001) among the most disadvantaged, 26.99 % (*p* < 0.001) among the middle 45 %, and 28.99 % among the least disadvantaged. The adjusted readmission rate was 29.42 % (*p* < 0.001) among the most disadvantaged, 32.70 % (*p* < 0.001) among the middle deprived, and 34.07 % among the least disadvantaged. Patients from the most disadvantaged communities experienced a 6.31 % greater adjusted rate of observation stay revisits compared to patients from the least disadvantaged communities.

## Discussion

In this cross-sectional, retrospective study of discharge data for patients with SMI from Florida and New York between 2012 and 2015, we showed that community socioeconomic disadvantage was significantly associated with revisits among patients with SMI even after adjusting for patient and hospital characteristics. This finding is consistent with other work supporting the impact of community socioeconomic disadvantage on health outcomes [11, 13, 21]. For example, Kind et al. found that residents of the most disadvantaged communities had higher rates of 30-day readmissions than residents of less disadvantaged communities [13]. Hu et al. extended the study by finding that patients residing in the most disadvantaged communities had significantly higher 30-day readmission risk compared to those in less disadvantaged communities even after accounting for individual-level factors (i.e. age, sex, race, marital status, primary diagnosis at the time of discharge, and comorbidities) [21].

The findings we found about the significant relationship between community socioeconomic disadvantage and revisits were consistent across all types of revisits (ED visits, observation stays, and readmissions) within 30-days of discharge. Patients from the most disadvantaged communities had over 3 % greater observation stay adjusted revisit rates than patients from the middle and least disadvantaged communities. Among the 497,243 patients who had a revisit over the time period, patients from the most disadvantaged communities had 30-day observation stay revisit rates 7.3 % points higher than the middle and least disadvantaged communities. They were less likely to experience a 30-day readmission or a 30-day ED visit. This could be explained as observation and ED visits are less costly to payers than inpatient admissions [48]. This finding was consistent with one study using 2013 Medicare claims, which found that low-income Medicare beneficiaries are at greater risk of high use of observation care [49].

One other study using 2013 Medicare claims found that low-income Medicare beneficiaries are at greater risk of high use of observation care [49]. Several factors could contribute to the higher adjusted observation stay revisits rates among patients from the most disadvantaged communities. First, prior work has demonstrated an association between poverty and high use of hospital services [50]. Second, the hospitals to which they are being readmitted may be trying to reduce readmissions by intentionally placing these patients under observation instead of readmitting them [51, 52]. Third, hospitals may be unintentionally admitting these patients as observation stays. Specifically, clinical practice patterns are changing so that patients admitted for acute reasons are admitted under observation or discharged from the ED instead of being admitted to inpatient units [53]. Lastly, more patients from disadvantaged communities might be returning for lower-acuity conditions that can be treated under observation without a hospital admission. Prior studies have found that chronic disease burden and black race—both reflective of patients in more disadvantaged communities—predicted higher use of observation services [54]. Patients in more disadvantaged communities likely have poorer access to primary care management and follow-up after their medical-surgical hospitalizations and need acute care services that do not necessitate a hospital readmission.

The findings from our study point to the critical importance of social factors in determining health outcomes for marginalized populations such as people with SMI and suggest future directions for improving health equity. These findings add to the social determinants of health framework, which suggests that the circumstances in which people live shape their health outcomes [52]. Sustained investments in delivery system innovations including health homes and collaborative care approaches that facilitate timely follow-up care after acute care discharge could help decrease disparities in outcomes including revisits. The Affordable Care Act provided funding for state Medicaid agencies to invest in health homes that coordinate physical, behavioral, and social services for Medicaid beneficiaries through the use of nurse care managers [55]. Additionally, collaborative care models embed mental health providers within primary care settings could help bridge the needs of these vulnerable patients [56]. Further research should investigate the impact of health homes and collaborative care on outcomes for individuals with SMI from disadvantaged communities.

## Limitations

The findings in this study are subject to limitations. First, the data used in this study are only from two states, so the findings reported here may not be generalizable to other communities or to other states. The ability to detect an effect of community socioeconomic disadvantage and the relationship between disadvantage and different types of revisits may differ across study contexts (e.g. different states). Therefore, further research is needed that examines the relationship between community socioeconomic disadvantage and revisits nationally. Second, even though a wide range of variables were considered in the analyses based on the existing literature, we were not able to completely disaggregate the effects of patient and hospital level characteristics given their complex interplay. We did not have access to patient race/ethnicity data for both states. Prior research has shown that patient race and ethnicity may interact with area deprivation in prediction poor outcomes [57]. We suggest future research examines the relationship between race/ethnicity and area deprivation. Third, we only had access to the five-digit ZIP codes of patients, so we were not able to use the more granular ADI ZIP + 4 characteristics.

## Conclusions

The results of this study strongly suggest that individuals with SMI from more disadvantaged communities have higher rates of 30-day revisits and specifically higher rates of 30-day observation stays. The ever-widening gap between advantaged and disadvantaged communities may exacerbate poor health outcomes. This poses questions about appropriate investments in disadvantaged communities to reduce disparities in health outcomes. Future work should compare the impact of socioeconomic disadvantage on outcomes of patients with SMI to those without SMI. Future work should also consider additional characteristics of the revisits, such as revisit diagnosis, which may help explain the differences in revisit type. Additionally, future work should investigate the effects of other community-level health investments such as availability of primary care clinics and federally qualified health centers on health outcomes like revisits.

## Supplementary Information

**Additional file 1.**

**Additional file 2.**

**Additional file 3.**

## Data Availability

Summary results generated or analyzed during this study are included in this published article and its supplementary information files. The raw data that supports the findings of this study is available for purchase from the Agency for Healthcare Research and Quality’s Healthcare Cost and Utilization Project (HCUP) at https://www.hcup-us.ahrq.gov/tech_assist/centdist.jsp, but restrictions apply to the availability of this data under their Data Use Agreement (DUA). The data used for this study was analyzed under a DUA with HCUP.

## Abbreviations

SMI: Serious Mental Illness
ED: Emergency Department
HCUP: Healthcare Cost and Utilization Project
AHRQ: Agency for Healthcare Research and Quality
SID: State Inpatient Database
SEDD: State Emergency Department Database
SASD: State Ambulatory Surgery and Services Database
LOS: Length of Stay
AHA: American Hospital Association
AHRF: Area Health Resources Files
ADI: Area Deprivation Index
ACS: American Community Survey
CCS: Clinical Classification Software
ICD: International Classification of Disease
