# Supplementary material for: Community socioeconomic disadvantage drives type of 30-day medical-surgical revisits among patients with serious mental illness
Source: BMC Health Serv Res. 2021 Jul 5;21:653. doi: 10.1186/s12913-021-06605-y (PMC8256502; doi:10.1186/s12913-021-06605-y)
Supplement: Supplementary file 1 — Additional file 1. [file 12913_2021_6605_MOESM1_ESM.docx]

Supplementary File 1. CCS Categories for Mental Illness, ICD-9 Codes for Planned Admissions, and CCS Categories for Planned Admissions

AHRQ CCS Categories for Serious Mental Illness

| 650 |
| --- |
| 651 |
| 652 |
| 654 |
| 655 |
| 656 |
| 657 |
| 658 |
| 659 |
| 663 |
| 670 |
| 660 |
| 661 |
| 663 |

ICD-9 Codes Used to Identify Planned Admissions

| \| 650 \| \| --- \| \| 65100 \| \| 65101 \| \| 65103 \| \| 65110 \| \| 65111 \| \| 65113 \| \| 65120 \| \| 65121 \| \| 65123 \| \| 65130 \| \| 65131 \| \| 65133 \| \| 65140 \| \| 65141 \| \| 65143 \| \| 65150 \| \| 65151 \| \| 65153 \| \| 65160 \| \| 65161 \| \| 65163 \| \| 65170 \| \| 65171 \| \| 65173 \| \| 65180 \| \| 65181 \| \| 65183 \| \| 65190 \| \| 65191 \| \| 65193 \| \| 65200 \| \| 65201 \| \| 65203 \| \| 65210 \| \| 65211 \| \| 65213 \| \| 65220 \| \| 65221 \| \| 65223 \| \| 65230 \| \| 65231 \| \| 65233 \| \| 67012 \| \| 67014 \| \| 67020 \| \| 67022 \| \| 67024 \| \| 67030 \| \| 67032 \| \| 67034 \| \| 67080 \| \| 67082 \| \| 67084 \| \|  \| | \| 65240 \| \| --- \| \| 65241 \| \| 65243 \| \| 65250 \| \| 65251 \| \| 65253 \| \| 65260 \| \| 65261 \| \| 65263 \| \| 65270 \| \| 65271 \| \| 65273 \| \| 65280 \| \| 65281 \| \| 65283 \| \| 65290 \| \| 65291 \| \| 65293 \| \| 65300 \| \| 65301 \| \| 65303 \| \| 65310 \| \| 65311 \| \| 65313 \| \| 65320 \| \| 65321 \| \| 65323 \| \| 65330 \| \| 65331 \| \| 65333 \| \| 65340 \| \| 65341 \| \| 65343 \| \| 65350 \| \| 65351 \| \| 65353 \| \| 65360 \| \| 65361 \| \| 65363 \| \| 65370 \| \| 65371 \| \| 65373 \| \| 65380 \| \|  \| | \| 65381  65383 \| \| --- \| \| 65390 \| \| 65391 \| \| 65393 \| \| 65400 \| \| 65401 \| \| 65402 \| \| 65403 \| \| 65404 \| \| 65410 \| \| 65411 \| \| 65412 \| \| 65413 \| \| 65414 \| \| 65420 \| \| 65421 \| \| 65423 \| \| 65430 \| \| 65431 \| \| 65432 \| \| 65433 \| \| 65434 \| \| 65440 \| \| 65441 \| \| 65442 \| \| 65443 \| \| 65444 \| \| 65450 \| \| 65451 \| \| 65452 \| \| 65453 \| \| 65454 \| \| 65460 \| \| 65461 \| \| 65462 \| \| 65463 \| \| 65464 \| \| 65470 \| \| 65471 \| \| 65472 \| \| 65473 \| \| 65474 \| \|  \| \|  \| | \| 65480 \| \| --- \| \| 65481 \| \| 65482 \| \| \| 65483 \| \| \| 65484 \| \| \| 65490 \| \| \| 65491 \| \| \| 65492 \| \| \| 65493 \| \| \| 65494 \| \| \| 65500 \| \| \| 65501 \| \| \| 65503 \| \| \| 65510 \| \| \| 65511 \| \| \| 65513 \| \| \| 65520 \| \| \| 65521 \| \| \| 65523 \| \| \| 65530 \| \| \| 65531 \| \| \| 65533 \| \| \| 65540 \| \| \| 65541 \| \| \| 65543 \| \| \| 65550 \| \| \| 65551 \| \| \| 65553 \| \| \| 65560 \| \| \| 65561 \| \| \| 65563 \| \| \| 65570 \| \| \| 65571 \| \| \| 65573 \| \| \| 65580 \| \| \| 65581 \| \| \| 65583 \| \| \| 65590 \| \| \| 65591 \| \| \| 65593 \| \| \| 65600 \| \| \| 65601 \| \| \| 65603 \| \| \|  \| \| \|  \| \| | \| 65610 \| \| --- \| \| 65611 \| \| 65613 \| \| 65620 \| \| 65621 \| \| 65623 \| \| 65630 \| \| 65631 \| \| 65633 \| \| 65640 \| \| 65641 \| \| 65643 \| \| 65650 \| \| 65651 \| \| 65653 \| \| 65660 \| \| 65661 \| \| 65663 \| \| 65670 \| \| 65671 \| \| 65673 \| \| 65680 \| \| 65681 \| \| 65683 \| \| 65690 \| \| 65691 \| \| 65693 \| \| 65700 \| \| 65701 \| \| 65703 \| \| 65800 \| \| 65801 \| \| 65803 \| \| 65810 \| \| 65811 \| \| 65813 \| \| 65820 \| \| 65821 \| \| 65823 \| \| 65830 \| \| 65831 \| \| 65833 \| \| 65840 \| \|  \| \|  \| | \| 65841 \| \| --- \| \| 65843 \| \| 65880 \| \| 65881 \| \| 65883 \| \| 65890 \| \| 65891 \| \| 65893 \| \| 65900 \| \| 65901 \| \| 65903 \| \| 65910 \| \| 65911 \| \| 65913 \| \| 65920 \| \| 65921 \| \| 65923 \| \| 65930 \| \| 65931 \| \| 65933 \| \| 65940 \| \| 65941 \| \| 65943 \| \| 65950 \| \| 65951 \| \| 65953 \| \| 65960 \| \| 65961 \| \| 65963 \| \| 65970 \| \| 65971 \| \| 65973 \| \| 65980 \| \| 65981 \| \| 65983 \| \| 65990 \| \| 65991 \| \| 65993 \| \| 66000 \| \| 66001 \| \| 66003 \| \| 66010 \| \| 66011 \| \|  \| \|  \| | \| 66013 \| \| --- \| \| 66021 \| \| 66023 \| \| 66030 \| \| 66031 \| \| 66033 \| \| 66040 \| \| 66041 \| \| 66043 \| \| 66050 \| \| 66051 \| \| 66053 \| \| 66060 \| \| 66061 \| \| 66063 \| \| 66070 \| \| 66071 \| \| 66073 \| \| 66080 \| \| 66081 \| \| 66083 \| \| 66090 \| \| 66091 \| \| 66093 \| \| 66100 \| \| 66101 \| \| 66103 \| \| 66110 \| \| 66111 \| \| 66113 \| \| 66120 \| \| 66121 \| \| 66123 \| \| 66130 \| \| 66131 \| \| 66133 \| \| 66140 \| \| 66141 \| \| 66143 \| \| 66190 \| \| 66191 \| \| 66193 \| \| 66200 \| \|  \| | 66201   \| 66203 \| \| --- \| \| 66210 \| \| 66211 \| \| 66213 \| \| 66220 \| \| 66221 \| \| 66223 \| \| 66230 \| \| 66231 \| \| 66233 \| \| 66300 \| \| 66301 \| \| 66303 \| \| 66310 \| \| 66311 \| \| 66313 \| \| 66320 \| \| 66321 \| \| 66323 \| \| 66330 \| \| 66331 \| \| 66333 \| \| 66340 \| \| 66341 \| \| 66343 \| \| 66350 \| \| 66351 \| \| 66353 \| \| 66360 \| \| 66361 \| \| 65363 \| \| 66380 \| \| 66381 \| \| 66383 \| \| 66390 \| \| 66391 \| \| 66393 \| \| 67000 \| \| 67002 \| \| 67004 \| \| 67010 \| |
| --- | --- | --- | --- | --- | --- | --- | --- | --- | --- | --- | --- | --- | --- | --- | --- | --- | --- | --- | --- | --- | --- | --- | --- | --- | --- | --- | --- | --- | --- | --- | --- | --- | --- | --- | --- | --- | --- | --- | --- | --- | --- | --- | --- | --- | --- | --- | --- | --- | --- | --- | --- | --- | --- | --- | --- | --- | --- | --- | --- | --- | --- | --- | --- | --- | --- | --- | --- | --- | --- | --- | --- | --- | --- | --- | --- | --- | --- | --- | --- | --- | --- | --- | --- | --- | --- | --- | --- | --- | --- | --- | --- | --- | --- | --- | --- | --- | --- | --- | --- | --- | --- | --- | --- | --- | --- | --- | --- | --- | --- | --- | --- | --- | --- | --- | --- | --- | --- | --- | --- | --- | --- | --- | --- | --- | --- | --- | --- | --- | --- | --- | --- | --- | --- | --- | --- | --- | --- | --- | --- | --- | --- | --- | --- | --- | --- | --- | --- | --- | --- | --- | --- | --- | --- | --- | --- | --- | --- | --- | --- | --- | --- | --- | --- | --- | --- | --- | --- | --- | --- | --- | --- | --- | --- | --- | --- | --- | --- | --- | --- | --- | --- | --- | --- | --- | --- | --- | --- | --- | --- | --- | --- | --- | --- | --- | --- | --- | --- | --- | --- | --- | --- | --- | --- | --- | --- | --- | --- | --- | --- | --- | --- | --- | --- | --- | --- | --- | --- | --- | --- | --- | --- | --- | --- | --- | --- | --- | --- | --- | --- | --- | --- | --- | --- | --- | --- | --- | --- | --- | --- | --- | --- | --- | --- | --- | --- | --- | --- | --- | --- | --- | --- | --- | --- | --- | --- | --- | --- | --- | --- | --- | --- | --- | --- | --- | --- | --- | --- | --- | --- | --- | --- | --- | --- | --- | --- | --- | --- | --- | --- | --- | --- | --- | --- | --- | --- | --- | --- | --- | --- | --- | --- | --- | --- | --- | --- | --- | --- | --- | --- | --- | --- | --- | --- | --- | --- | --- | --- | --- | --- | --- | --- | --- | --- | --- | --- | --- | --- | --- | --- | --- | --- | --- | --- | --- | --- | --- | --- | --- | --- | --- | --- | --- | --- | --- | --- | --- | --- | --- | --- | --- | --- | --- | --- | --- | --- | --- | --- | --- | --- | --- | --- | --- | --- | --- | --- | --- | --- | --- | --- | --- | --- | --- | --- | --- | --- | --- | --- | --- | --- | --- | --- | --- | --- | --- | --- | --- | --- | --- | --- | --- | --- | --- | --- | --- | --- | --- | --- | --- | --- | --- | --- | --- | --- | --- | --- | --- | --- | --- | --- | --- | --- | --- | --- | --- | --- | --- | --- | --- | --- | --- | --- | --- | --- |

AHRQ CCS categories used to identify planned admissions

| DXCCS Codes:   \| 11 \| \| --- \| \| 12 \| \| 13 \| \| 14 \| \| 15 \| \| 16 \| \| 17 \| \| 18 \| \| 19 \| \| 20 \| \| 21 \| \| 23 \| \| 24 \| \| 25 \| \| 26 \| \| 27 \| \| 28 \| \| 29 \| \| 30 \| \| 31 \| \| 32 \| \| 33 \| \| 34 \| \| 35 \| \| 36 \| \| 37 \| \| 38 \| \| 39 \| \| 40 \| \| 41 \| \| 42 \| \| 43 \| \| 44 \| \| 45 \| \| 194 \| \| 196 \| \| 254 \| | PRCCS Codes:   \| 64 \| \| --- \| \| 105 \| \| 134 \| \| 135 \| \| 176 \| |
| --- | --- | --- | --- | --- | --- | --- | --- | --- | --- | --- | --- | --- | --- | --- | --- | --- | --- | --- | --- | --- | --- | --- | --- | --- | --- | --- | --- | --- | --- | --- | --- | --- | --- | --- | --- | --- | --- | --- | --- | --- | --- | --- | --- |
