# Supplementary material for: Community socioeconomic disadvantage drives type of 30-day medical-surgical revisits among patients with serious mental illness
Source: BMC Health Serv Res. 2021 Jul 5;21:653. doi: 10.1186/s12913-021-06605-y (PMC8256502; doi:10.1186/s12913-021-06605-y)
Supplement: Supplementary file 2 — Additional file 2. [file 12913_2021_6605_MOESM2_ESM.docx]

Supplementary File 2: Primary Reasons for Index Admission, by Community Disadvantage

|  | All patients | Least Disadvantaged | Middle 45% Disadvantaged | Top 5% Disadvantaged | p-value |
| --- | --- | --- | --- | --- | --- |
| **Primary Reason for Index admission (N, %)** |  |  |  |  |  |
| Cardiac | 342,467, 17.9% | 174,484, 18.2% | 153,239, 17.7% | 14,744, 16.2% | <0.001 |
| Nervous System | 137,981, 7.2% | 70,004, 7.3% | 61,066, 7.1% | 6,911, 7.6% | <0.001 |
| Cardiac Obstructive Pulmonary Disease or Asthma | 134,558, 7.0% | 59,402, 6.2% | 68,476, 7.9% | 6,680, 7.3% | <0.001 |
| Alcohol or Drug | 109,115, 5.7% | 64,367, 6.7% | 38,412, 4.4% | 6,336, 7.0% | <0.001 |
| Renal Failure | 85,372, 4.5% | 41,007, 4.28% | 40,101, 4.6% | 4,264, 4.7% | <0.001 |
| Kidney Transplant, renal failure/dialysis | 32,743, 1.7% | 14,962, 1.6% | 15,798, 1.8% | 1,983, 2.2% | <0.001 |
| Cerebrovascular | 48,958, 2.6% | 23,801, 2.5% | 22,668, 2.6% | 2,489, 2.7% | <0.001 |
| GI Hemorrhage | 40,507, 2.1% | 20,404, 2.1% | 18,233, 2.1% | 1,870, 2.1% | 0.212 |
| Diabetes | 27,407, 1.4% | 12,082, 1.3% | 13,346, 1.5% | 1,979, 2.2% | <0.001 |
| Liver | 24,574, 1.3% | 12,007, 1.3% | 11,453, 1.3% | 1,114, 1.2% | <0.001 |
| Nutrition/metabolic | 22,584, 1.2% | 11,808, 1.2% | 9,776, 1.1% | 1,000, 1.1% | <0.001 |
| Obesity | 18,499, 1.0% | 9,590, 1.0% | 7,923, 0.9% | 986, 1.1% | <0.001 |
| Anemia | 18,001, 0.9% | 8,422, 0.9% | 8,411 1.0% | 1,168, 1.3% | <0.001 |
| Peripheral Vascular | 15,595, 0.8% | 7,625, 0.8% | 7,222, 0.8% | 748, 0.8% | 0.015 |
| Hypertensive, Uncomplicated | 10,076, 0.5% | 4,815, 0.5% | 4,658, 0.5% | 603, 0.7% | <0.001 |
| Thyroid/Endocrine | 6,094, 0.3% | 3,076, 0.3% | 348, 0.4% | 6,094, 0.3% | 0.001 |
| Human Immunodeficiency Virus | 3,719, 0.2% | 917, 0.1% | 2,412, 0.3% | 390, 0.4% | <0.001 |
| Connective Tissue | 3,018, 0.2% | 1,496, 0.2% | 1,342, 0.2% | 180, 0.20% | 0.008 |
| Coagulation Disorders | 1,651, 0.1% | 917, 0.1% | 664, 0.1% | 70, 0.1% | <0.001 |
| Hypertensive, Complicated | 1,514, 0.1% | 647, 0.1% | 743, 0.1% | 124, 0.1% | <0.001 |
