# Supplementary material for: Community socioeconomic disadvantage drives type of 30-day medical-surgical revisits among patients with serious mental illness
Source: BMC Health Serv Res. 2021 Jul 5;21:653. doi: 10.1186/s12913-021-06605-y (PMC8256502; doi:10.1186/s12913-021-06605-y)
Supplement: Supplementary file 3 — Additional file 3. [file 12913_2021_6605_MOESM3_ESM.docx]

**Supplementary File 3. Full Regression Models**

1. **Logistic Regression Models for Odds of Any Type of Revisit within 30 Days**

|  | **Patient Covariates** | | | **Patient + Hospital** | | | **Patient + Hospital + Community** | | |
| --- | --- | --- | --- | --- | --- | --- | --- | --- | --- |
|  | OR | 95% CI | p-value | OR | 95% CI | p-value | OR | 95% CI | p-value |
| Area Deprivation Index |  |  |  |  |  |  |  |  |  |
| Least Disadvantaged | Reference | | | | | | | | |
| Middle 45% Disadvantaged | 1.02 | 1.01-1.03 | <0.001 | 1.02 | 1.01-1.03 | .001 | 1.02 | 1.01-1.03 | .002 |
| Top 5% Disadvantaged | 1.03 | 1.00-1.05 | .010 | 1.03 | 1.00-1.05 | .037 | 1.05 | 1.02-1.07 | <0.001 |
| Age |  |  |  |  |  |  |  |  |  |
| <=44 | Reference | | | | | | | | |
| 45-64 | 1.00 | .99-1.02 | .727 | 1.01 | .99-1.02 | .448 | 1.00 | .99-1.02 | .509 |
| 65+ | .90 | .88-.91 | <0.001 | .90 | .87-.92 | <0.001 | .90 | .89-.92 | <0.001 |
| Female | .96 | .95-.97 | <0.001 | .96 | .95-.97 | <0.001 | .96 | .95-.97 | <0.001 |
| Payer |  |  |  |  |  |  |  |  |  |
| Medicare | Reference | | | | | | | | |
| Medicaid | .99 | .97-1.00 | .105 | .98 | .97-1.00 | .037 | .99 | .97-1.01 | .198 |
| Private | .74 | .73-.75 | <0.001 | .74 | .73-.75 | <0.001 | .74 | .73-.76 | <0.001 |
| Self-Pay | .66 | .64-.67 | <0.001 | .66 | .64-.67 | <0.001 | .65 | .63-.66 | <0.001 |
| No Charge | .74 | .71-.77 | <0.001 | .75 | .71-.78 | <0.001 | .73 | .70-.76 | <0.001 |
| Other | .80 | .78-.82 | <0.001 | .80 | .77-.82 | <0.001 | .80 | .77-.82 | <0.001 |
| Length of Stay |  |  |  | 1.00 | .997-.999 | <0.001 | 1.00 | 997-1.00 | <0.001 |
| Admission Type |  |  |  |  |  |  |  |  |  |
| Emergent | Reference | | | | | | | | |
| Urgent |  |  |  | 1.00 | .98-1.02 | .971 | 1.00 | .98-1.02 | .844 |
| Elective |  |  |  | .81 | .79-.82 | <0.001 | .81 | .79-.82 | <0.001 |
| Trauma |  |  |  | .97 | .90-1.04 | .373 | .96 | .89-1.04 | .302 |
| OR Procedure |  |  |  | .93 | .92-.95 | <0.001 | .93 | .92-.94 | <0.001 |
| Readmission Risk Score |  |  |  | 1.02 | 1.015-1.016 | <0.001 | 1.02 | 1.015-1.016 | <0.001 |
| DRGs |  |  |  |  |  |  |  |  |  |
| Cardiac |  |  |  | 1.24 | 1.22-1.25 | <0.001 | 1.24 | 1.22-1.25 | <0.001 |
| Kidney Transplant, renal failure/dialysis |  |  |  | 1.17 | 1.14-1.21 | <0.001 | 1.17 | 1.14-1.21 | <0.001 |
| Renal Failure |  |  |  | 1.11 | 1.06-1.16 | <0.001 | 1.11 | 1.06-1.16 | <0.001 |
| Nervous System |  |  |  | .98 | .95-1.01 | .119 | .98 | .95-1.00 | .109 |
| Cerebrovascular |  |  |  | .83 | .79-.87 | <0.001 | .83 | .79-.87 | <0.001 |
| Peripheral Vascular |  |  |  | 1.32 | 1.25-1.39 | <0.001 | 1.31 | 1.25-1.38 | <0.001 |
| Cardiac Obstructive Pulmonary Disease or Asthma |  |  |  | 1.16 | 1.14-1.18 | <0.001 | 1.16 | 1.13-1.18 | <0.001 |
| Diabetes |  |  |  | 1.17 | 1.12-1.22 | <0.001 | 1.17 | 1.12-1.22 | <0.001 |
| Liver |  |  |  | 1.77 | 1.71-1.84 | <0.001 | 1.77 | 1.70-1.83 | <0.001 |
| Ulcer |  |  |  | 1.07 | 1.03-1.11 | <0.001 | 1.07 | 1.03-1.10 | <0.001 |
| Human Immunodeficiency Virus |  |  |  | 1.35 | 1.22-1.49 | <0.001 | 1.31 | 1.18-1.44 | <0.001 |
| Connective Tissue |  |  |  | 1.80 | 1.63-2.00 | <0.001 | 1.80 | 1.63-1.99 | <0.001 |
| Nutrition/metabolic |  |  |  | 1.15 | 1.10-1.20 | <0.001 | 1.15 | 1.10-1.21 | <0.001 |
| Anemia |  |  |  | 1.46 | 1.39-1.53 | <0.001 | 1.46 | 1.39-1.53 | <0.001 |
| Alcohol or Drug |  |  |  | 1.17 | 1.15-1.20 | <0.001 | 1.19 | 1.16-1.21 | <0.001 |
| Coagulation Disorders |  |  |  | 1.70 | 1.48-1.95 | <0.001 | 1.70 | 1.47-1.96 | <0.001 |
| Hypertensive, complicated |  |  |  | 1.00 | .84-1.20 | .968 | 1.00 | .84-1.20 | .977 |
| Hypertensive, uncomplicated |  |  |  | .82 | .76-.89 | <0.001 | .82 | .75-.88 | <0.001 |
| Obesity |  |  |  | .94 | .88-1.00 | .070 | .96 | .90-1.02 | .211 |
| Teaching Hospital |  |  |  | 1.06 | 1.04-1.07 | <0.001 | 1.07 | 1.05-1.09 | <0.001 |
| Total Number of Beds |  |  |  | 1.00 | 1<0.00106-1<0.00109 | <0.001 | 1.00 | 1<0.00104-1<0.00107 | <0.001 |
| High Technology Status |  |  |  | .99 | .98-1.01 | .240 | .98 | .97-.99 | .005 |
| Nurse to Bed Ratio |  |  |  | 1.01 | 1.00-1.03 | .010 | 1.02 | 1.01-1.03 | .004 |
| Hospital Ownership |  |  |  |  |  |  |  |  |  |
| Public | Reference | | | | | | | | |
| Private, Not for profit |  |  |  | .97 | .96-.99 | .001 | .99 | .97-1.01 | .230 |
| Private, For profit |  |  |  | 1.06 | 1.04-1.08 | <0.001 | 1.03 | 1.01-1.05 | .005 |
| Rural |  |  |  |  |  |  | 1.01 | .9997-1.0239 | .057 |
| Physicians per person (in county) |  |  |  |  |  |  | 1.00 | 1<0.00110-1<0.00119 | <0.001 |
| Nurse Practitioners per person (in county) |  |  |  |  |  |  | 1.00 | .998879-.999372 | <0.001 |
| State |  |  |  |  |  |  |  |  |  |
| Florida | Reference | | | | | | | | |
| New York |  |  |  |  |  |  | .84 | .082-.088 | <0.001 |

1. **Logistic Regression Models for Odds of Emergency Department Revisit within 30 Days**

|  | **Patient Covariates** | | | **Patient + Hospital** | | | **Patient + Hospital + Community** | | |
| --- | --- | --- | --- | --- | --- | --- | --- | --- | --- |
|  | OR | 95% CI | p-value | OR | 95% CI | p-value | OR | 95% CI | p-value |
| Area Deprivation Index |  |  |  |  |  |  |  |  |  |
| Least Disadvantaged | Reference | | | | | | | | |
| Middle 45% Disadvantaged | 1.20 | 1.19-1.22 | <0.001 | 1.8 | 1.6-1.19 | <0.001 | 1.08 | 1.07-1.09 | <0.001 |
| Top 5% Disadvantaged | 1.58 | 1.13-1.18 | <0.001 | 1.15 | 1.12-1.17 | <0.001 | 1.09 | 1.07-1.02 | <0.001 |
| Age |  |  |  |  |  |  |  |  |  |
| <=44 | Reference | | | | | | | | |
| 45-64 | .72 | .71-.73 | <0.001 | .72 | .71-.73 | <0.001 | .72 | .72-.73 | <0.001 |
| 65+ | .49 | .48-.50 | <0.001 | .49 | .48-.50 | <0.001 | .49 | .49-.50 | <0.001 |
| Female | 1.06 | 1.05-1.07 | <0.001 | 1.05 | 1.04-1.06 | <0.001 | 1.05 | 1.04-1.06 | <0.001 |
| Payer |  |  |  |  |  |  |  |  |  |
| Medicare | Reference | | | | | | | | |
| Medicaid | 1.11 | 1.09-1.13 | <0.001 | 1.13 | 1.11-1.15 | <0.001 | 1.18 | 1.16-1.20 | <0.001 |
| Private | .68 | .67-.70 | <0.001 | .69 | .68-.70 | <0.001 | .70 | .69-.71 | <0.001 |
| Self-Pay | 1.12 | 1.10-1.14 | <0.001 | 1.11 | 1.09-1.13 | <0.001 | 1.08 | 1.06-1.10 | <0.001 |
| No Charge | 1.24 | 1.19-1.28 | <0.001 | 1.21 | 1.17-1.25 | <0.001 | 1.14 | 1.0-1.18 | <0.001 |
| Other | .99 | .97-1.02 | .569 | .99 | .97-1.02 | .633 | .98 | .95-1.00 | .060 |
| Length of Stay |  |  |  | .97 | .970-.973 | <0.001 | .97 | .971-.974 | <0.001 |
| Admission Type |  |  |  |  |  |  |  |  |  |
| Emergent |  | | | | | | | | |
| Urgent |  |  |  | 1.30 | 1.28-1.32 | <0.001 | 1.25 | 1.23-1.27 | <0.001 |
| Elective |  |  |  | .76 | .75-.78 | <0.001 | .75 | .74-.77 | <0.001 |
| Trauma |  |  |  | 2.49 | 2.38-2.61 | <0.001 | 2.38 | 2.27-2.50 | <0.001 |
| OR Procedure |  |  |  | 1.12 | 1.11-1.14 | <0.001 | 1.12 | 1.10-1.13 | <0.001 |
| Readmission Risk Score |  |  |  | 1.01 | 1.0060-1.0069 | <0.001 | 1.01 | 1.0056-1.0067 | <0.001 |
| DRGs |  |  |  |  |  |  |  |  |  |
| Cardiac |  |  |  | 1.06 | 1.05-1.08 | <0.001 | 1.07 | 1.06-1.09 | <0.001 |
| Kidney Transplant, renal failure/dialysis |  |  |  | 1.06 | 1.03-1.09 | <0.001 | 1.06 | 1.02-1.09 | <0.001 |
| Renal Failure |  |  |  | .91 | .87-.96 | <0.001 | .91 | .87-.96 | <0.001 |
| Nervous System |  |  |  | 1.40 | 1.37-1.43 | <0.001 | 1.41 | 1.38-1.44 | <0.001 |
| Cerebrovascular |  |  |  | .89 | .86-.93 | <0.001 | .89 | .86-.92 | <0.001 |
| Peripheral Vascular |  |  |  | 1.37 | 1.30-1.43 | <0.001 | 1.37 | 1.30-1.43 | <0.001 |
| Cardiac Obstructive Pulmonary Disease or Asthma |  |  |  | .92 | .90-.94 | <0.001 | .91 | .89-.93 | <0.001 |
| Diabetes |  |  |  | .89 | .86-.93 | <0.001 | .90 | .86-.94 | <0.001 |
| Liver |  |  |  | .91 | .87-.95 | <0.001 | .90 | .86-.94 | <0.001 |
| Ulcer |  |  |  | .91 | .88-.95 | <0.001 | .91 | .88-.95 | <0.001 |
| Human Immunodeficiency Virus |  |  |  | .92 | .83-1.02 | .124 | .85 | .77-.95 | .002 |
| Connective Tissue |  |  |  | 1.00 | .89-1.13 | .986 | 1.01 | .90-1.14 | .852 |
| Nutrition/metabolic |  |  |  | 1.08 | 1.03-1.13 | .001 | 1.09 | 1.04-1.14 | <0.001 |
| Anemia |  |  |  | .81 | .76-.85 | <0.001 | .81 | .77-.86 | <0.001 |
| Alcohol or Drug |  |  |  | 1.56 | 1.53-1.59 | <0.001 | 1.67 | 1.63-1.70 | <0.001 |
| Coagulation Disorders |  |  |  | .80 | .67-.96 | .016 | .81 | .68-.97 | .024 |
| Hypertensive, complicated |  |  |  | .87 | .73-1.03 | .101 | .86 | .72-1.02 | .085 |
| Hypertensive, uncomplicated |  |  |  | 1.09 | 1.02-1.16 | .010 | 1.10 | 1.03-1.17 | .004 |
| Obesity |  |  |  | .78 | .74-.82 | <0.001 | .82 | .77-.87 | <0.001 |
| Teaching Hospital |  |  |  | .89 | .87-.90 | <0.001 | 1.00 | .98-1.02 | .936 |
| Total Number of Beds |  |  |  | 1.00 | .99997-.99999 | .010 | 1.00 | .99994-99997 | <0.001 |
| High Technology Status |  |  |  | 1.04 | 1.03-1.05 | <0.001 | .97 | .98-1.00 | .029 |
| Nurse to Bed Ratio |  |  |  | 1.02 | 1.00-1.03 | .004 | 1.01 | 1.00-1.02 | .175 |
| Hospital Ownership |  |  |  |  |  |  |  |  |  |
| Public | Reference | | | | | | | | |
| Private, Not for profit |  |  |  | .99 | .97-1.01 | .223 | 1.03 | 1.01-1.04 | .001 |
| Private, For profit |  |  |  | 1.08 | 1.06-1.10 | <0.001 | .97 | .95-.99 | <0.001 |
| Rural |  |  |  |  |  |  | 1.06 | 1.05-1.08 | <0.001 |
| Physicians per person (in county) |  |  |  |  |  |  | 1.00 | .9993-.9994 | <0.001 |
| Nurse Practitioners per person (in county) |  |  |  |  |  |  | 1.00 | 1.00193-1.002401 | <0.001 |
| State |  |  |  |  |  |  |  |  |  |
| Florida | Reference | | | | | | | | |
| New York |  |  |  |  |  |  | .79 | .78-.79 | <0.001 |

1. **Logistic Regression Models for Odds of Observation Stay Revisit within 30 Days**

|  | **Patient Covariates** | | | **Patient + Hospital** | | | **Patient + Hospital + Community** | | |
| --- | --- | --- | --- | --- | --- | --- | --- | --- | --- |
|  | OR | 95% CI | p-value | OR | 95% CI | p-value | OR | 95% CI | p-value |
| Area Deprivation Index |  |  |  |  |  |  |  |  |  |
| Least Disadvantaged | Reference | | | | | | | | |
| Middle 45% Disadvantaged | .83 | .82-.84 | <0.001 | 1.01 | 1.00-1.02 | .042 | 1.32 | 1.30-1.34 | <0.001 |
| Top 5% Disadvantaged | 1.35 | 1.32-1.38 | <0.001 | 1.54 | 1.51-1.58 | <0.001 | 1.46 | 1.43-1.50 | <0.001 |
| Age |  |  |  |  |  |  |  |  |  |
| <=44 | Reference | | | | | | | | |
| 45-64 | 1.14 | 1.13-1.16 | <0.001 | 1.15 | 1.13-1.17 | <0.001 | 1.14 | 1.13-1.16 | <0.001 |
| 65+ | .93 | .91-.95 | <0.001 | .94 | .92-.96 | <0.001 | .90 | .88-.92 | <0.001 |
| Female | .94 | .93-.95 | <0.001 | .96 | .95-.97 | <0.001 | .98 | .96-.99 | <0.001 |
| Payer |  |  |  |  |  |  |  |  |  |
| Medicare | Reference | | | | | | | | |
| Medicaid | 1.40 | 1.38-1.43 | <0.001 | 1.28 | 1.26-1.30 | <0.001 | 1.10 | 1.08-1.12 | <0.001 |
| Private | .88 | .87-.90 | <0.001 | .84 | .83-.86 | <0.001 | .79 | .77-.80 | <0.001 |
| Self-Pay | .54 | .52-.55 | <0.001 | .56 | .54-.58 | <0.001 | .68 | .6-.70 | <0.001 |
| No Charge | .20 | .18-.21 | <0.001 | .21 | .19-.23 | <0.001 | .43 | .39-.47 | <0.001 |
| Other | .69 | .67-.71 | <0.001 | .68 | .66-.70 | <0.001 | .71 | .69-.74 | <0.001 |
| Length of Stay |  |  |  | .98 | .9801-.9830 | <0.001 | .98 | .9754-.9783 | <0.001 |
| Admission Type |  |  |  |  |  |  |  |  |  |
| Emergent | Reference | | | | | | | | |
| Urgent |  |  |  | 1.12 | 1.10-1.14 | <0.001 | 1.27 | 1.24-1.30 | <0.001 |
| Elective |  |  |  | .77 | .76-.79 | <0.001 | .80 | .79-.82 | <0.001 |
| Trauma |  |  |  | .78 | .71-.85 | <0.001 | .93 | .85-1.02 | .141 |
| OR Procedure |  |  |  | .96 | .95-.98 | <0.001 | .98 | .96-.99 | .009 |
| Readmission Risk Score |  |  |  | 1.00 | .9993-1.002 | .320 | 1.00 | 1<0.001-1.001 | .002 |
| DRGs |  |  |  |  |  |  |  |  |  |
| Cardiac |  |  |  | 1.03 | 1.02-1.05 | <0.001 | 1.00 | .98-1.01 | .860 |
| Kidney Transplant, renal failure/dialysis |  |  |  | 1.63 | 1.58-1.67 | <0.001 | 1.70 | 1.65-1.75 | <0.001 |
| Renal Failure |  |  |  | .65 | .62-.68 | <0.001 | .65 | .62-.68 | <0.001 |
| Nervous System |  |  |  | .92 | .90-.95 | <0.001 | .91 | .88-.93 | <0.001 |
| Cerebrovascular |  |  |  | .99 | .95-1.04 | .767 | 1.01 | .97-1.06 | .604 |
| Peripheral Vascular |  |  |  | 1.16 | 1.09-1.23 | <0.001 | 1.21 | 1.14-1.28 | <0.001 |
| Cardiac Obstructive Pulmonary Disease or Asthma |  |  |  | .71 | .69-.73 | <0.001 | .73 | .71-.75 | <0.001 |
| Diabetes |  |  |  | 1.03 | .98-1.07 | .216 | .99 | .95-1.03 | .640 |
| Liver |  |  |  | 1.72 | 1.66-1.79 | <0.001 | 1.77 | 1.70-1.84 | <0.001 |
| Ulcer |  |  |  | 1.04 | 1.00-1.08 | .038 | 1.06 | 1.02-1.10 | .002 |
| Human Immunodeficiency Virus |  |  |  | .14 | .11-.19 | <0.001 | .34 | .25-.46 | <0.001 |
| Connective Tissue |  |  |  | 1.45 | 1.29-1.62 | <0.001 | 1.42 | 1.27-1.60 | <0.001 |
| Nutrition/metabolic |  |  |  | .93 | .89-.98 | .006 | .89 | .84-.94 | <0.001 |
| Anemia |  |  |  | 1.42 | 1.35-1.49 | <0.001 | 1.42 | 1.35-1.49 | <0.001 |
| Alcohol or Drug |  |  |  | .59 | .57-.61 | <0.001 | .47 | .46-.49 | <0.001 |
| Coagulation Disorders |  |  |  | 1.63 | 1.41-1.90 | <0.001 | 1.59 | 1.37-1.86 | <0.001 |
| Hypertensive, complicated |  |  |  | .89 | .73-1.10 | .276 | .94 | .76-1.15 | .540 |
| Hypertensive, uncomplicated |  |  |  | .89 | .82-.96 | .003 | .87 | .81-.95 | .001 |
| Obesity |  |  |  | 1.11 | 1.05-1.17 | <0.001 | .83 | .79-.88 | <0.001 |
| Teaching Hospital |  |  |  | 1.67 | 1.65-1.70 | <0.001 | 1.07 | 1.05-1.09 | <0.001 |
| Total Number of Beds |  |  |  | 1.00 | .9997-.9998 | <0.001 | 1.00 | .999995-1<0.00103 | .187 |
| High Technology Status |  |  |  | .66 | .65-.67 | <0.001 | .90 | .88-.91 | <0.001 |
| Nurse to Bed Ratio |  |  |  | .99 | .98-1.00 | .014 | 1.00 | .99-1.01 | .463 |
| Hospital Ownership |  |  |  |  |  |  |  |  |  |
| Public | Reference | | | | | | | | |
| Private, Not for profit |  |  |  | .85 | .84-.86 | <0.001 | .63 | .62-.64 | <0.001 |
| Private, For profit |  |  |  | .26 | .25-.26 | <0.001 | .66 | .64-68 | <0.001 |
| Rural |  |  |  |  |  |  | 1.04 | 1.03-1.05 | <0.001 |
| Physicians per person (in county) |  |  |  |  |  |  | 1.00 | 1<0.0011-1<0.0012 | <0.001 |
| Nurse Practitioners per person (in county) |  |  |  |  |  |  | 1.00 | .9993-.9998 | <0.001 |
| State |  |  |  |  |  |  |  |  |  |
| Florida | Reference | | | | | | | | |
| New York |  |  |  |  |  |  | 5.36 | 5.26-5.45 | <0.001 |
